# Impact of Unplanned Excision on Prognosis of Patients with Extremity Soft Tissue Sarcoma

**DOI:** 10.1155/2013/498604

**Published:** 2013-04-30

**Authors:** Hafiz Muhammad Umer, Masood Umer, Irfan Qadir, Nadeem Abbasi, Nehal Masood

**Affiliations:** ^1^Department of Surgery, Section of Orthopaedic Surgery, Aga Khan University Hospital, Stadium Road, Karachi 74800, Pakistan; ^2^Department of Oncology, Section of Radiation Oncology, Aga Khan University Hospital, Stadium Road, Karachi 74800, Pakistan; ^3^Department of Oncology, Aga Khan University Hospital, Stadium Road, Karachi 74800, Pakistan

## Abstract

Unplanned excision of soft tissue sarcomas (STSs) outside comprehensive tumor management centers necessitates the need for wide reexcision to achieve adequate margins. We retrospectively reviewed medical records of 135 patients with STS operated at our hospital with the goal of examining outcomes, in terms of local recurrence (LR) and metastasis rate (MR), of reexcision following unplanned excision of STS and comparing results with those of first-time planned surgery. Eighty-four patients had their first-time surgery and 51 patients had come to us following unplanned excision at prereferral hospital. Mean age of all patients was 41.8 ± 21.9 years. The LR and MR was 14.3% and 8.3%, respectively, in patients undergoing first resection, whereas it was 21.4% and 13.7%, respectively, in patients undergoing revision surgery. Average duration from previous unplanned excision was 8 months. Twelve patients were referred immediately after excised specimen revealed STS, while 39 patients presented after evident local recurrence. Wide reexcision was attempted in 48 patients while three patients need amputation. Adjuvant radiotherapy was administered in all patients undergoing limb-sparing surgery. Ten patients needed adjuvant chemotherapy. We conclude that wide reexcision of STS has poorer outcomes compared to planned excision. Therefore, patients with soft tissue masses should be managed by multidisciplinary oncology team at specialized cancer centers.

## 1. Introduction

Soft tissue sarcomas (STSs) are heterogeneous groups of malignant mesenchymal tumors which comprise less than 1% of all malignancies [1]. The literature has reported that a general physician may see one STS case in 24 years [2]. Meticulous workup in terms of radiological and histopathological evaluation is required for diagnosis and planning a multidisciplinary treatment strategy. The standard treatment protocol involves wide surgical resection along with neoadjuvant or adjuvant radiotherapy [3]. 

In a developing country like ours, health care system is still immature. Patients with limb swellings seek treatment very late and often doctors are their last choice. Many end up with general surgeons who still prefer to excise these without any knowledge of proper margins [4]. Moreover, due to rarity of soft tissue sarcomas coupled with low index of suspicion for malignancy, these soft tissue masses are not evaluated properly by radiological and histopathological examination by general orthopedic surgeons. The patients diagnosed with STS each year in USA are approximately 8700, whereas in U.K, about 1500 new STS cases are diagnosed [5]. Bhurgri et al. [6] reported that STS in Pakistan accounted for 2.9% and 1.6% of all cancers in males and females, respectively. They also reported that although STS does not fall in 10 most common cancers of the region, Pakistan still falls into high risk category for STS with male predominance [6]. In a country of 180 million people, we only have three trained orthopedic oncology surgeons. Consequently, such masses are excised by inexperienced surgeons without consideration of malignancy. The risk of residual disease after such unplanned excision is reported to be between 24%–60% in the literature [7–12].

Patients with extremity lumps *n* bumps end up in an excision biopsy with the intent of removing a benign tumor. They are then referred to specialized tumor care centers when the pathology of excised specimen reveals an STS or when there is local recurrence. At this stage, various treatment options can be offered to the patient. These include wide re-resection followed by adjuvant therapy, radiotherapy alone or amputation. Most studies favor wide re-resection along with radiotherapy in a specialized tumor care center [10, 13, 14].

The purpose of this study was to examine the outcomes of reexcision of residual STSs following an unplanned excision and compare it, in terms of local recurrence and metastasis, with outcomes of patients who underwent a single, planned excision.

## 2. Patients and Methods

We conducted this retrospective cohort study at The Aga Khan Hospital on consecutive patients coming to our hospital from 1994 to 2008. The demographic data and clinical characteristics of the study population were acquired from clinical chart review, tumor registry information, physicians' records, patients' correspondence, and telephone interviews. Patients were excluded if they had incomplete or missing medical records. A total of 135 patients were thus included in the study.

Patients were divided into two groups. The “planned excision” group consisted of 84 patients who were referred to orthopedic oncologists at our institution without any prior attempts at excision of tumor. The tumor was then excised after proper staging workup and intraoperative frozen section were done to confirm the diagnosis and margin status. The “unplanned excision” group consisted of 51 patients who were referred after a previous attempt to excise the tumor at some other hospital. Reexcision was carried out for these patients unless the tumor invasion was extensively involving the neurovascular structures. Intraoperative pathologist was consulted to confirm the negative margins and diagnosis.

All tumors were reviewed by experienced pathologists at our institution. Tumors were diagnosed and graded according to the FNCLCC (French Federation Nationale des Centres de Lutte Contre le Cancer) system [15]. For analyses, FNCLCC grade 2 and 3 tumors were defined as high-grade tumors, and grade 1 as a low grade one [16]. Tumor size was classified as <5 cm or ≥5 cm. Tumors were characterized as superficial or deep according to the involvement of the investing fascia [17]. Margins were evaluated intraoperatively by a dedicated pathologist. Margins were inked and separately sampled. The closest margin was microscopically categorized as positive (tumor within 1 mm of the inked surface) or negative (absence of tumor within 1 mm of the inked surface) and was further classified into the following categories: <4 mm, 5–9 mm, 10–19 mm, and ≥20 mm. Radiotherapy was recommended for patients with tumors exhibiting high risk factors for recurrence: any one or combination of factors including size >5 cm, high grade, deep tumors, inadequate surgical margins. Radiation therapy was given to the entire surgical bed with 3 to 5 cm margin beyond the surgical scar and/or beyond post-operative seroma or areas of ecchymoses. The dose delivered was 50 Gy in 25 fractions using computed tomography (CT-) based three-dimensional conformal treatment planning. The scar was bolused to ensure full dose to the surface. The field size was then reduced to the primary surgical bed plus a 2 cm margin for a further dose of 10 Gy in 5 fractions. If the margin was microscopically positive, a further 6 Gy in 3 fractions was delivered to that area for a total of 66 Gy in 33 fractions.

Statistical software SPSS version 20 was used for data analysis. Chi square was used for categorical variables and *t* test was used for continuous variables. A *P* value of <0.05 was considered to indicate statistical significance.

## 3. Results

During the study period, a total of 135 patients received treatment at our institute. Mean age of the patients was 41.8 ± 21.9 years. There were 83 (61.5%) men and 52 (38.5%) women. Histological diagnosis of tumor was made by taking biopsies as recommended by NCCN guidelines. pleomorphic liposarcoma, synovial cell sarcoma, spindle cell sarcoma, and malignant fibrous histiocytoma were the most common tumor types. The majority of the tumors were located in the lower limb (67.4%). The clinical pathological characteristics of patients are summarized in Table 1. 

## 4. Group 1: Planned Excision

There were 84 patients in this group. Most of the patients presented with asymptomatic swelling. The mean duration of symptoms was 12 months. Most of the tumors were of high grade (84.5%), ≥5 cm in size (59.5%), and deep seated (44%). Eighteen tumors (21.4%), 28 (33.3%), 34 (40.5%), and 4 (4.8%) were categorized as having margin widths of 1–4 mm, 5–9 mm, 10–19 mm, and ≥20 mm, respectively. Postoperative radiation was administered to 60 patients. Patients with prognostic factors indicating high risk for recurrence were recommended for radiation therapy.

The mean follow-up period (starting from the date of surgery) was 52.6 ± 39.8 months (median 36 months). As shown in Table 2, twelve patients (14.3%) developed local recurrence. Distant metastasis occurred in 7.3% of the patients. Sixty-five patients were alive in this group at the end of the study while 8 patients died during the followup.

## 5. Group 2: Unplanned Excision

There were 51 patients in this group. Average duration from previous unplanned excision to wide reexcision was 8 months. 12 patients were referred immediately after excised specimen revealed STS, while 39 patients presented after evident local recurrence. Wide reexcision was attempted in 48 patients while three patients needed amputation. Adjuvant radiotherapy was administered in all patients undergoing limb sparing surgery. Additionally ten patients needed adjuvant chemotherapy. Most of the tumors in this group were of high grade (84.3%), deep seated (50.9%), and <5 cm in size (70.5%).

Minimum follow-up duration (starting from revision surgery) was 36 months. As shown in Table 2, 11 (21.4%) patients developed local re-recurrence. Distant metastasis occurred in 7 (13.7%) of the patients. 

## 6. Discussion

The term “unplanned total excision” was first described by Giuliano and Eilber [7]. Noria et al. [8] further described this term with respect to soft tissue sarcoma as unplanned resection of a lesion without using benefits of preoperative diagnostic modalities and without intention to achieve tumor-free margins. The literature has suggested that such resections result from the lack of awareness of standard soft tissue sarcoma management and can cause of problem when potential malignancy is not considered [3, 18]. 

Soft tissue sarcomas have low incidence and can occur in any part of the body. This leads physicians other than surgical oncologists to resect these tumors with non-standardized methods and without keeping a suspicion of malignancy in mind [3, 18]. 

In order to avoid such unplanned resections, physicians must be aware of the signs reported by the literature which suggest malignancy. These signs include mass size >5 cm, rapid increase in size of the mass, presence of mass deep to fascia, pain in previously painless mass, and recurrent swelling [3, 19]. The literature has reported several diagnostic modalities such as biopsy, proper staging, and preoperative imaging. These must be carried out in specialized sarcoma centers. These modalities not only help in diagnosis but are also of importance in avoiding incorrect surgical procedures on these sarcomas [3, 18].

Pakistan is a third world country and its population is different from others in terms of access to health centers, awareness about the disease, and socioeconomic status. There are only a few specialized cancer care centers in the country with multidisciplinary approach towards these rare STSs. Patients therefore end up going to improper health care personnel and getting the lesion excised without keeping in mind the suspicion of malignancy and safe margins. By the time they approach the specialized cancer care centers, they have a poor prognosis. The presence of a multidisciplinary tumor board is an integral part of multimodal therapy for malignant diseases, particularly for sarcoma [4].

Once an unplanned excision has been carried out, there is a need for reexcision by an experienced surgical oncologist. The literature has also reported that surgical margins need to be more extensive during reexcision as compared to conventional wide-margin excision because CT/MRI shows reactive changes post-operatively after first surgery and inappropriate skin incisions lead to contamination of neighboring compartments. The purpose is to remove the residual tumor and the neighboring tissue which was contaminated during unplanned excision [3, 20]. Considering the large number of cases with residual disease found in re-excised specimen there is a need of single-planned surgery. Residual tumors in re-excised specimen are associated with increased local recurrence and metastasis [3].

The published literature has reported variable outcomes when studying prognostic outcomes of patients who have received the unplanned resection.  Table 3 shows comparison of the number of cases with residual tumor in various studies. Lewis et al. [12] showed that disease specific metastasis-free survival rate was lower in patients who underwent re-resection as compared to those who underwent planned primary surgery. Fiore et al. [21] reported that there were no significant differences in terms of local relapse, metastasis, and mortality between the reexcision group and the primary planned surgery group. Ueda et al. [22] furthermore showed that patients who received initial inadequate excision had higher local recurrence rates as compared to those who had their planned primary definitive surgery at the same institution. Our study reported that local recurrence rate, metastasis rate, and mortality rate were higher in patients who underwent unplanned resections, although the difference as compared to patients who underwent planned primary surgery was statistically insignificant.

The loss of statistical significance between the outcomes of the groups could be explained by selection and referral bias. Smaller and superficial masses are more likely to be excised by nonspecialist surgeon on the basis of assumption that they are benign. Conversely, large and deep-seated tumors which have potential worse prognosis are referred to a cancer care center for the planned primary excision. However, in our data, there was no statistical difference between the baseline characteristics of the two groups (as detailed in Table 1). Therefore, worse outcome of unplanned excision group could not be attributed to the aggressiveness of tumors.

Although re-resection is an outcome to unplanned surgeries, it has its own costs. Patient's affected limb function may be reduced and complicated after re-resection as compared to primary planned surgery due to the surgical approach used during the re-resection. Secondly, unplanned surgery usually leads to contamination of neighboring compartments with the tumor cells so it becomes very difficult to ensure the complete removal of cancerous cells in reexcision. Furthermore, the need for re-resection as early as possible leads to sparing little time for neoadjuvant therapy which plays an important part in the management of sarcoma patient [23].

## 7. Conclusion

Although the difference in rates of local recurrence and metastasis between the two groups are statistically insignificant, a second surgery is translated into greater morbidity in terms of pain, functional class of patient, aggressiveness, technical difficulty of second surgery, prolonged length of hospital stay, and cost of treatment as compared to planned primary surgery. Therefore patients with soft-tissue masses of unknown identity should be appropriately imaged, biopsied, and managed by orthopedic oncologist at a specialized tumor centre. 

## Figures and Tables

**Table 1:** Clinical characteristics of patients.

Characteristics	Total *N* = 135, No. (%)	Planned surgery *N* = 84, No. (%)	Unplanned surgery				*P* value
			All *N* = 51	Immediate *N* = 12	Delayed *N* = 39	*P* value	
Age							0.523
<45	75 (55.5%)	47 (56.0%)	28 (54.9%)	6 (50%)	22 (56.4%)	0.696	
≥45	60 (44.5%)	37 (44.0%)	23 (45.1%)	6 (50%)	17 (43.6%)		
Gender							0.039
Male	83 (61.5%)	46 (54.8%)	37 (72.5%)	9 (75%)	28 (71.8%)	0.828	
Female	52 (38.5%)	38 (45.2%)	14 (27.5%)	3 (25%)	11 (28.2%)		
Tumor site							0.207
Upper	44 (32.6%)	25 (29.8%)	19 (37.3%)	2 (16.7%)	17 (43.6%)	0.092	
Lower	91 (67.4%)	59 (70.2%)	32 (62.7%)	10 (83.3%)	22 (56.4%)		
Grade							0.152
1	21 (15.5%)	13 (15.5%)	8 (15.7%)	4 (33.3%)	4 (10.3%)	0.158	
2	42 (31.1%)	31 (36.9%)	11 (21.6%)	2 (16.7%)	9 (23%)		
3	72 (53.4%)	40 (47.6%)	32 (62.7%)	6 (50%)	26 (66.7%)		
Size							0.001
<5 cm	70 (51.8%)	34 (40.5%)	36 (70.5%)	12 (100%)	24 (61.5%)	0.011	
>5 cm	65 (48.2%)	50 (59.5%)	15 (29.5%)	0 (0%)	15 (38.5%)		
Radio							0.000
Yes	95 (70.3%)	47 (71.4%)	48 (94.1%)	11 (91.7%)	37 (94.9%)	0.680	
No	40 (29.7%)	37 (28.6%)	3 (5.9%)	1 (8.3%)	2 (5.1%)		
Margin							0.447
1–4 mm	29 (21.5%)	18 (21.4%)	11 (21.6%)	5 (41.7%)	6 (15.4%)	0.203	
5–9 mm	39 (28.9%)	28 (33.3%)	11 (21.6%)	1 (8.3%)	10 (25.6%)		
10–19 mm	58 (42.9%)	34 (40.5%)	24 (47.1%)	6 (50%)	18 (46.2%)		
>20	6 (4.4%)	4 (4.8%)	2 (3.9%)	0 (0%)	2 (5.1%)		
Depth							0.504
Superficial	72 (53.4%)	47 (56.0%)	25 (49.9%)	9 (75%)	16 (41.1%)	0.040	
Deep	63 (46.6%)	37 (44.0%)	26 (50.9%)	3 (25%)	23 (58.9%)		

**Table 2:** compares the outcomes of two groups.

Patients' outcomes	Total *N* = 135, No. (%)	Planned surgery *N* = 84, No. (%)	Unplanned surgery *N* = 51, No. (%)	*P* value
Recurrence				0.275
Yes	23 (17%)	12 (14.3%)	11 (21.5%)	
No	112 (83%)	72 (85.7%)	40 (78.5%)	
Metastasis				0.319
Yes	14 (10%)	7 (8.3%)	7 (13.7%)	
No	121 (90%)	77 (91.7%)	44 (86.3%)	
Status				0.679
Dead	15 (11%)	8 (9.5%)	7 (13.7%)	
Alive	120 (89%)	76 (91.5%)	44 (86.3%)	

**Table 3:** Comparison the number of cases with residual tumors in various studies.

Study	Total cases	Reexcision (%)	Residual tumor (%)
Noria et al. [8]	65	100	35
Davis et al. [9]	239	43	40
Fiore et al. [21]	597	53	24
Chandrasekar et al. [10]	363	87	60
Venkatesan et al. [3]	42	92.5	74
Our study	51	100	68.6
